# Uncovering novel MHC alleles from RNA-Seq data: expanding the spectrum of MHC class I alleles in sheep

**DOI:** 10.1186/s12863-022-01102-5

**Published:** 2023-01-03

**Authors:** Johannes Buitkamp

**Affiliations:** Bavarian State Research Center for Agriculture, Institute of Animal Breeding, 85586 Grub, Germany

**Keywords:** RNA-Seq, Sheep, MHC class I, Novel alleles, Ovar-N, BWA-MEM, cap3

## Abstract

**Background:**

Major histocompatibility complex (MHC) class I glycoproteins present selected peptides or antigens to CD8 + T cells that control the cytotoxic immune response. The MHC class I genes are among the most polymorphic loci in the vertebrate genome, with more than twenty thousand alleles known in humans. In sheep, only a very small number of alleles have been described to date, making the development of genotyping systems or functional studies difficult. A cost-effective way to identify new alleles could be to use already available RNA-Seq data from sheep. Current strategies for aligning RNA-Seq reads against annotated genome sequences or transcriptomes fail to detect the majority of class I alleles. Here, I combine the alignment of RNA-Seq reads against a specific reference database with de novo assembly to identify alleles. The method allows the comprehensive discovery of novel MHC class I alleles from RNA-Seq data (DinoMfRS).

**Results:**

Using DinoMfRS, virtually all expressed MHC class I alleles could be determined. From 18 animals 75 MHC class I alleles were identified, of which 69 were novel. In addition, it was shown that DinoMfRS can be used to improve the annotation of MHC genes in the sheep genome sequence.

**Conclusions:**

DinoMfRS allows for the first time the annotation of unknown, more divergent MHC alleles from RNA-Seq data. Successful application to RNA-Seq data from 16 animals has approximately doubled the number of known alleles in sheep. By using existing data, alleles can now be determined very inexpensively for populations that have not been well studied. In addition, MHC expression studies or evolutionary studies, for example, can be greatly improved in this way, and the method should be applicable to a broader spectrum of other multigene families or highly polymorphic genes.

**Supplementary Information:**

The online version contains supplementary material available at 10.1186/s12863-022-01102-5.

## Introduction

Major histocompatibility complex (MHC) class I genes encode cell surface glycoproteins expressed on all nucleated cells without marked tissue or cell specificity. MHC class I molecules present short (8–11 amino acids) peptides derived from proteolysis of intracellular proteins to CD8 + T cells, natural killer cells and myeloid cells [1–3]. The set of peptides that is presented by the receptors of an organism, organ or cell is referred to as the MHC ligandome/peptidome or immunopeptidome [4]. The binding of a specific MHC/peptide complex to a specific T cell receptor (Tcr) regulates the mechanisms of host defense by triggering a rapid CD8 + T cell response after infection, the recognition of cancer cells and self *vs.* non-self recognition by negative selection of self-reactive T cells. This dedicated functional role explains the numerous associations of MHC alleles with disease resistance e.g. in human [5, 6] or farm animals [7–10] as well as autoimmune diseases e.g. Morbus Bechterew [11], rheumatoid arthritis [12], or type 1 diabetes [13].

To ensure sufficient recognition of the plethora of potential antigens at the population level MHC class I genes are highly polymorphic. In particular, this concerns the amino acid positions that are part of the antigen binding groove and bind the antigen (anchor residues) and those in direct contact with the Tcr molecules (mediating the so called self restriction). These positions, in the antigen-binding groove and the Tcr contact region are located in the α1 and α2 domain of the MHC-I heavy chain. These regions are fully encoded by exons 2 and 3 of the MHC class I genes, which are therefore highly polymorphic, whereas the 3' region of the sequence is more conserved. At the individual level a sufficient number of different MHC alleles is obtained by heterozygosity (most individuals are heterozygote at the MHC) and by being polygenic, i.e. more than one MHC class I gene per haplotype exists (“heterozygosity across multiple loci”). Accordingly, in most vertebrate populations a very high number of class I alleles exist. In humans more than 20.000 alleles are described [14]. The genomic organization of the human classical MHC class I genes is uniform, i.e. the region consistently carry three highly polymorphic genes *(HLA-A, -B, -C)* per haplotype [15].

In sheep, only a very small number [32 IPD curated alleles, 16] of class I alleles are known to date. These are deposited in the immuno polymorphism database (IPD). IPD contains, among others, the MHC alleles for sheep and cattle. It is the official repository and main source for manually curated sequence data and allele nomenclature [16, 17]. Moreover, the overall genomic organization of the genes is not yet clear. In contrast to humans the number of ovine class I genes is known to vary between haplotypes, but very few haplotypes have been described. These contain 2–3 class I genes [18–20]. In cattle, the closest relative of sheep with significant information about MHC class I genes (IPD lists 127 classical and 51 non-classical alleles) a larger number of (IPD lists 10 curated) haplotypes is described, but even in this species the information is sparse. Some haplotypes that were assumed to be well characterized had to be extended by an additional allele that was previously overlooked by several studies. Haplotype A14, for example was initially described to contain three expressed class I genes [21], whereas meanwhile it could be shown to contain four genes [22]. The most comprehensive study in cattle describes 1–4 classical Class I genes per haplotype with 2 at highest frequency and 22 haplotypes derived from analyzing SRA data [23].

The limited number of ovine alleles known and the lack of information about the genomic organization complicate the detection of new alleles and the development of efficient genotyping systems. Genotyping of MHC genes is further complicated by multiple heterozygous positions (hindering the definition of alleles from direct sequencing), 0-alleles, and the potential co-amplification of different genes by PCR-based methods. In human and some model organism robust methods based on the well characterized allelic spectrum had been established (e.g. [24]). Different methods of MHC genotyping in less characterized species had been proposed, from a combination of PCR and hybridization with specific oligonucleotides to next generation sequencing (NGS) [25–27]. Nevertheless, the reliability of these typing methods depends largely on the knowledge of haplotype structure and a comprehensive library of alleles.

Therefore expanding and completing the panel of known alleles is a crucial step in the development of MHC typing systems in sparsely characterized populations. In former times the most common method was the cloning and sequencing of PCR products to obtain the sequences of single alleles in farm animals (e.g. [28, 29]). Currently primarily NGS methods are used. For example, in cattle MHC class I genes were amplified using two specific PCR-systems that were based on previous knowledge about potential alleles. PCR products were analyzed using the Illumina MiSeq platform [30]. This method proved to be effective in cattle and many previously unknown alleles and haplotypes were identified. For the application of this method in sheep, sufficient characterization of alleles and suitable PCR systems still need to be developed.

One way to increase the number of known class I alleles is to use already available next generation sequencing (NGS) data as e.g. from the European Nucleotide Archive database (ENA, [31]). In particular, as the number of RNA-Seq and whole-genome shotgun sequencing (WGS) datasets in sheep continues to increase, they represent an already available source to expand the allele database for MHC. Till now the high degree of polymorphism and the variable number of MHC class I genes per haplotype hinder the mapping of sequence reads obtained by NGS technology to a reference genome or transcriptome and current strategies for aligning RNA-Seq data to these sequences fail to identify the majority of class I alleles.

**Fig. 1:**
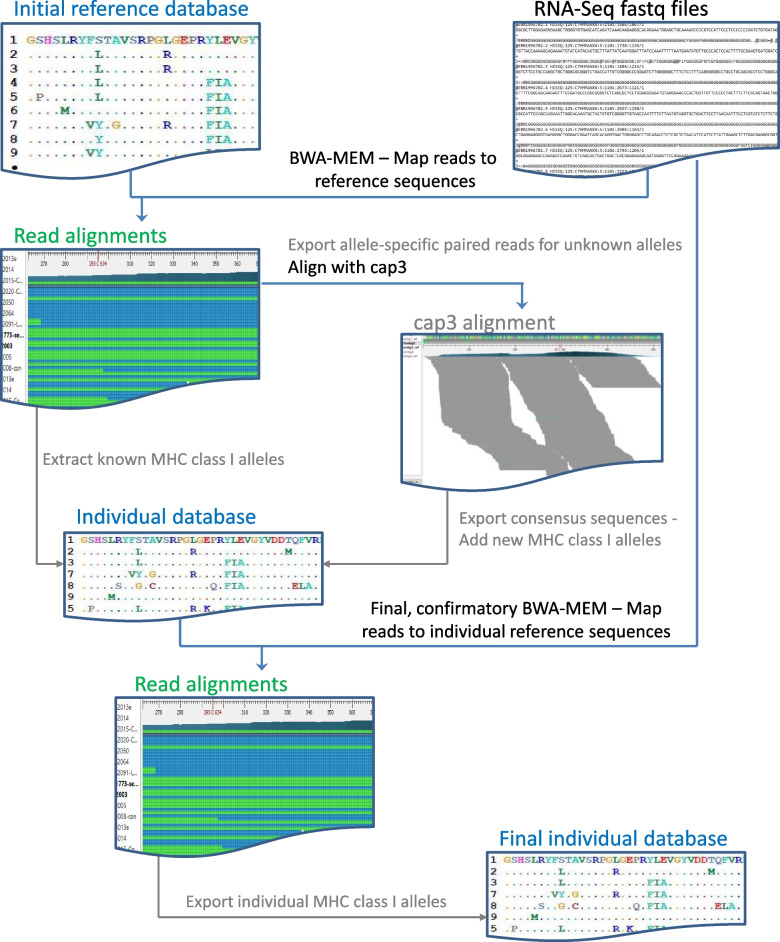
Workflow of Uncovering novel MHC alleles from RNA-Seq data (DinoMfRS). DinoMfRS enables the identification of previously unknown variant MHC alleles from RNA-Seq data through the creation of individual allele databases and a two-step approach that combines alignment of RNA-Seq reads to reference sequences and de novo assembly. The initial reference database (top left) consists of all known sheep MHC class I alleles. RNA-Seq reads (top right) are aligned to the class I reference sequences (using BWA-MEM). Known alleles carried by each individual show complete coverage, and the aligned RNA reads show no mismatches. From alignments with high coverage but mismatches to the reference allele (possibly novel alleles) RNA-Seq reads are extracted and de novo assembled using cap3 for further analysis. Consensus sequences from full-length cap3 assemblies are exported. An intermediate reference database containing the individual alleles will be created from these potentially novel alleles and the previously known alleles. A final BWA-MEM run is used to verify the novel alleles. The individual alleles of the examined sheep then result from the alignments showing complete coverage without mismatches

Here, I developed a method that enables the use of publicly available RNA-Seq data to define ovine MHC class I alleles. The method allows the discovery of novel MHC alleles from RNA-Seq data (DinoMfRS) and is based on alignment of RNA-Seq data to a limited initial MHC class I reference database created from the few known sheep sequences followed by de novo reassembly of a subset of RNA-Seq reads (align—> extraction of RNA-Seq reads—> reassemble, Fig. 1).

## Results

### Reference database

Thirty two ovine MHC class I sequences (the complete set of curated alleles from IPD) were retrieved from the IPD database, aligned and trimmed to the region of exons two and three. Two alleles (Ovar-N*03:02:01, Ovar-N*21:01) were highly similar or identical to other alleles and were deleted. From the NCBI nucleotide collection 19 additional sequences were added that were not covered by IPD and differed in ≥ 5 bp from each other allele. The initial reference database finally consisted of 49 ovine class I sequences.

### BWA-MEM alignments

The RNA-Seq data from 15 sheep were analyzed one by one. When new alleles were discovered from an individual these were added to the reference database facilitating step by step the analysis since the coverage of the allelic spectrum by the database improved. Alleles that matched those from IPD or from the NCBI nucleotide collection were termed according to the current allele nomenclature (“N*##:##”) or database accession number, respectively. New alleles were provisionally termed “LfL####”.

**Fig. 2:**
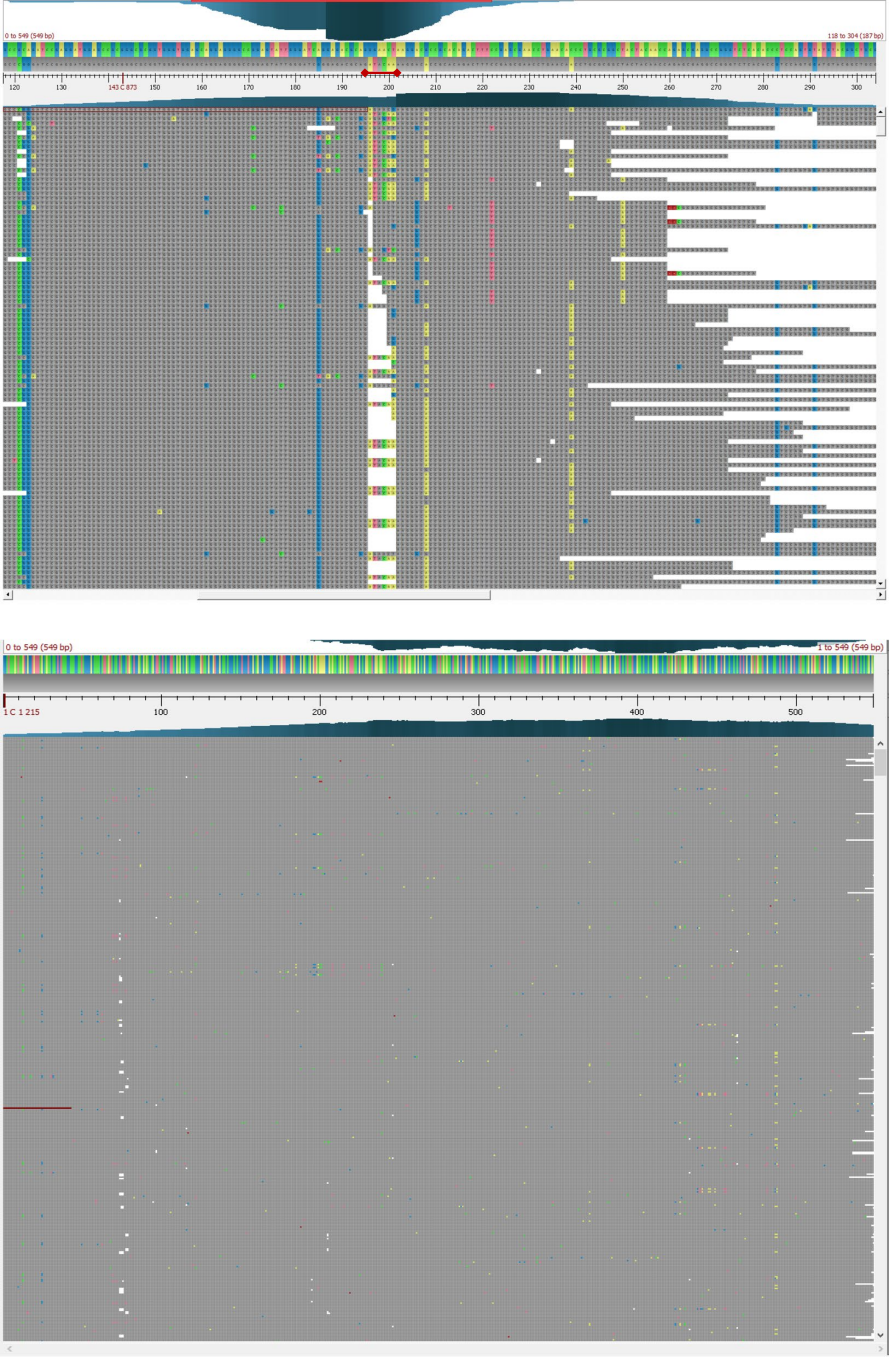
Visualization of reads aligned to MHC class I alleles using BWA-MEM. Obtaining new class I alleles from RNA-Seq data by successive BWA-MEM/cap3 runs (DinoMfRS) using animal 01 and allele LfL2003 as an example. Caption of BWA-MEM alignment results from the initial (top: alignment of the RNA-Seq reads to the reference allele N*06:01, the range from nt 256 to 264, which is different from all known sheep alleles, is indicated by a red line) and final (bottom: alignment against the new allele LfL2003) BWA-MEM run. Identity to reference is shown in gray, differences are shown in color (G—blue, A—yellow, T—red, C—green)

The initial DinoMfRS analysis for the first animal resulted in 6 alleles, all but one (Acc. U03094.1) so far unknown (LfL2001, LfL2003, LfL2006, LfL2015, LfL2037; Tables 1 and 2). Therefore, 5 alleles had to be analyzed by iterative steps and cap3 de novo alignment to identify the correct sequences. In the following, the identification of allele LfL2003 is described as it represents one of the alleles with the lowest similarity to any known sequence represented in the reference library (Table 1). The extraction of allele LfL2003 was further complicated by a nucleotide motif -CAGATACAA- at nucleotide position 256 to 264 (Fig. 2, positions according to full CDS from IPD) corresponding to amino acid sequence -QIQ- at positions 86 to 88 aa that was not described so far and differs in 4–8 from 9 nt from all known alleles in sheep. Until now it was only known in class I alleles from *sus scrofa* (e.g. SLA-1*16:03). The initial BWA-MEM run resulted in more than four thousand (K) reads aligned to the sequence Ovar N*13:02, and a large number of reads aligned to part of Ovar N*06:01, but several heterogeneous nucleotide positions showed up (the extracted consensus sequence showed only 514/549 bp identities to the final allele LfL2003). The cap3 alignment of these reads enabled the identification of one homogenous consensus sequence. The final BWA-MEM run against the individual database for sheep 01 containing all 6 alleles showed a homogenous alignment of reads to allele LfL2003 (Fig. 2). The addition of this allele to the reference library facilitated the analysis of animals 02 and 03 since they also carried this allele; the successive addition of further alleles speeded up the analysis of the next animals step by step.

**Table 1:** Ovine MHC class I allele from 16 individuals

Name	Acc.ver^a^	Identity	Species	Ovar-^b^	Identity	Transcription^c^	Class^d^	n^e^
U03094.1	U03094.1	100,0%	oa	N*18:01	95,6%	**	cl	3/0
LfL2003	XM_018038884.1	94,0%	ch	N*06:01	92,8%	**	cl	3/0
LfL2005	DQ121186.1	95,3%	bt	N*13:02	95,1%	**	cl	2/0
JQ824375.1	JQ824375.1	100,0%	oa	N*14:01	94,2%	**	cl	1/0
N*11:01	GQ150751.1	100,0%	oa	N*11:01	100,0%	**	cl	1/0
LfL2011	U03094.1	96,4%	oa	N*05:01	95,4%	**	cl	2/0
LfL2012	AJ874675.2	98,0%	oa	N*07:01	98,0%	**	cl	1/0
LfL2072	LT984572.1	99,3%	oa	N*12:01	99,3%	**	cl	1/0
LfL2014	EF489538.1	93,4%	oa	N*10:01	93,4%	***	cl	1/0
LfL2016	KC733413.1	98,4%	oa	N*17:01	98,4%	***	cl	1/0
LfL2020	NM_001308452.1	96,5%	oa	N*05:01	96,5%	***	cl	0/3
LfL2021	U03092.1	96,7%	oa	N*01:01	91,6%	**	cl	0/4
LfL2022	LT984575.1	94,5%	oa	N*26:01	94,0%	**	cl	0/4
LfL2024	LT984561.1	98,9%	oa	N*24:01	93,3%	**	cl	0/2
LfL2025	KX858769.1	99,8%	oa	N*11:01	94,5%	***	cl	0/1
LfL2026	KC733413.1	95,6%	oa	N*17:01	95,4%	**	cl	0/1
LfL2027	AM181175.1	96,0%	oa	N*02:01	99,8%	**	cl	0/2
LfL2028	NM_001130934.1	95,1%	oa	N*11:01	95,1%	***	cl	0/1
LfL2031	AJ874679.2	95,8%	oa	N*04:01	95,8%	***	cl	1/1
LT984558.1	LT984558.1	100,0%	oa	N*10:01	93,3%	***	cl	0/1
LfL2034a	EF489513.1	98,9%	oa	N*26:01	92,9%	***	cl	0/1
LfL2034b	EF489513.1	99,0%	oa	N*26:01	93,1%	***	cl	0/1
LfL2035	EF489519.1	97,3%	oa	N*20:01	95,4%	***	cl	0/1
LfL2037	KC733418.1	94,9%	oa	N*13:02	94,9%	***	cl	1/1
LfL2041	KX858768.1	97,6%	oa	N*20:01	97,3%	***	cl	0/1
LfL2044	LT984575.1	93,3%	oa	N*26:01	92,7%	**	cl	0/1
LfL2047	U03092.1	99,1%	oa	N*26:01	92,3%	**	cl	0/1
LfL2052	LT984576.1	92,7%	oa	N*27:01	92,7%	**	cl	1/0
LfL2054	KX858769.1	94,7%	oa	N*04:01	94,0%	**	cl	1/0
LfL2055	LT984572.1	98,9%	oa	N*12:01	98,9%	**	cl	1/0
LfL2059	EF569216.1	93,1%	ch	N*05:01	93,1%	**	cl	0/1
LfL2064	XM_018038832.1	94,9%	ch	N*10:01	84,8%	*	cl	0/1
LfL2043	EF569216.1	94,7%	ch	N*05:01	94,2%	**	nc	0/1
LfL2004	JQ031569.1	93,2%	bt	N*25:01	93,1%	**	nc	2/0
LfL2001	NM_001308586.1	98,7%	oa	N*50:03	98,7%	*	nc	1/2
LfL2013a	U03093.1	99,8%	oa	N*50:03	99,3%	*	nc	1/0
U03093.1	U03093.1	100,0%	oa	N*50:03	99,5%	*	nc	0/1
LfL2013e	NM_001308586.1	99,0%	oa	N*50:03	99,8%	*	nc	0/1
LfL2023	NM_001308586.1	99,6%	oa	N*50:03	98,4%	*	nc	0/2
N*50:01	AJ874677.2	100,0%	oa	N*50:01	100,0%	*	nc	0/1
LfL2057	NM_001308586.1	99,0%	oa	N*50:03	99,1%	*	nc	0/1
LfL2060	AJ874677.2	99,2%	oa	N*50:01	99,2%	*	nc	1/0
LfL2006	KX858770.1	92,6%	oa	N*19:01	92,2%	*	nc	3/0
LfL2008	LT984572.1	98,7%	oa	N*12:01	98,7%	*	nc	1/0
LfL2015	XM_027958893.1	99,6%	oa	N*20:01	90,5%	*	nc	3/2
LfL2017	KX858770.1	99,6%	oa	N*24:01	97,6%	*	nc	1/0
LfL2036	XM_027958913.1	94,4%	oa	N*13:02	91,8%	*	nc	0/1
LfL2046a	XM_027958903.1	96,6%	oa	N*06:01	91,3%	*	nc	0/1
LfL2046b	XM_027958903.1	97,1%	oa	N*14:01	89,3%	*	nc	0/1
LfL2051	JQ031569.1	93,1%	oa	N*04:01	93,1%	*	nc	1/0
LfL2058	LT984572.1	99,1%	oa	N*12:01	99,1%	*	nc	0/1
LfL2082	XM_027958893.2	99,8%	oa	N*20:01	90,1%	*	nc	ra
LfL2083	KX858769.1	96,7%	oa	N*11:01	96,7%	***	cl	ra
LfL2084	EF489513.1	96,8%	oa	N*26:01	92,5%	***	cl	ra
LfL2085	KX858763.1	97,5%	oa	N*12:01	97,3%	**	cl	ra
LfL2086	KC733416.1	96,6%	oa	N*20:01	96,5%	***	cl	ra
LfL2087	XM_042237435.1	100,0%	oa	N*06:01	91,4%	*	nc	ra
LfL2088	XM_018038836.1	97,8%	oa	N*07:01	93,6%	*	nc	ra
LfL2089	XM_042237461.1	100,0%	oa	N*50:02	98,0%	**	nc	ra
LfL2090	XM_042237461.1	98,7%	oa	N*50:02	94,9%	**	nc	ra
LfL2091	DQ121186.1	97%	oa	N*27:01	94,2%	***	cl	hu
LfL2092	KX858764.1	93%	oa	N*25:01	93,4%	***	nc	hu
LfL2093	NM_001308586.1	99%	oa	N*50:03	99,1%	*	nc	hu
LfL2094	EF489538.1	94%	oa	N*10:01	94,5%	***	cl	hu
LfL2095	EF489537.1	97%	oa	N*09:01	93,1%	**	cl	hu
LfL2096	EF489516.1	99%	oa	N*10:01	85,8%	*	nc	hu
LfL2097	XM_027958908.2	99%	oa	N*07:01	93%	*	nc	cr
LfL2098	OL628782.1	94%	oa	N*13:02	89%	***	cl	cr
LfL2099	OL628783.1	95%	oa	N*25:01	92%	***	cl	cr
LfL2100	AJ874678.2	99%	oa	N*50:02	99%	*	nc	cr
LfL2101	XM_018038824.1	98%	ch	no find	ns	*	nc	cr
LfL2102	OL628814.1	93%	oa	N*19:01	92%	**	nc	cr
LfL2103	AJ874674.2	96%	oa	N*01:01	95%	***	cl	cr
LfL2104	AJ874682.2	93%	oa	N*50:00	93%	**	nc	cr
LfL2105	OL628806.1	99%	oa	N*50:03	98%	**	nc	cr

^a^Accession number and version of the sequence with the highest score from the BLAST search against the Nucleotide collection (nt) at the NCBI followed by the identity in percent and the species (oa, *ovis aries*; bt*, bos taurus*; ch, *capra hircus*

^b^Closest or identical ovine MHC class I allele from the IPD MHC database with the highest identity score followed by the identity in percent

^c^Transcription level had been estimated from the number of reads aligned at position 260 bp (* < 1000, ** 1000–5000, *** > 5000 reads per allele)

^d^Preliminary classification: cl, classical; nc, putative non-classical

^e^Number of animals carrying the allele (Texel x Scottish Blackface crossbreed/Merino); ra, Rambouillet (Benz2616); hu, Husheep;

**Table 2:** MHC class I alleles per individual

Sheep	Classical MHC class I alleles						Putative non-classical MHC class I alleles				
	1	2	3	4	5	6	1	2	3	4	5
**01**	U03094.1	LfL2037	LfL2003				LfL2001	LfL2006	LfL2015		
**02**	U03094.1	JQ824375.1	LfL2003				LfL2001	LfL2006	LfL2004	LfL2008	
**03**	U03094.1	LfL2031	LfL2003				LfL2006	LfL2004	LfL2015	LfL2051	LfL2060
**04**	LfL2005	LfL2011	N*11:01				LfL2013e				
**05**	LfL2012	LfL2072	LfL2014	LfL2016	LfL2052	LfL2055	LfL2013a	LfL2015			
**06**	LfL2005	LfL2011	LfL2054				LfL2015	LfL2017			
**07**	LfL2021	LfL2022	LfL2020	LfL2059			LfL2057	LfL2064			
**08**	LfL2021	LfL2022	LfL2024				LfL2013a	LfL2023	LfL2058	LfL2046a	
**09**	LfL2025	LfL2026					LfL2015				
**10**	LfL2028	LfL2027					LfL2064	LfL2057	LfL2015		
**11**	LfL2035	LfL2031	LfL2034a				U03093	LfL2036			
**12**	LfL2021	LfL2022	LfL2034b	LfL2037	LfL2041		LfL2001	LfL2015	LfL2046b		
**13**	LfL2020	LfL2059	LfL2027				LfL2043	LfL2023	LfL2015		
**14**	LfL2020	LfL2059	LfL2047	LfL2044			U03093	LfL2046b	LfL2015		
**15**	LfL2021	LfL2022	LT984558				LfL2013e	N*50:01	LfL2008		
**16**	LfL2083	LfL2084	LfL2085	LfL2086			LfL2082	LfL2087	LfL2088	LfL2089	LfL2090
**17**	LfL2091	LfL2094	LfL2095	N*11:01			LfL2092	LfL2093	LfL2096		
**18**	LfL2098	LfL2099	LfL2102	LfL2103			LfL2097	LfL2100	LfL2101	LfL2104	LfL2105

MHC class I alleles identified by DinoMfRS from Texel x Scottish Blackface crossbreed (01–06), from Merino sheep (07–15), one Rambouillet (16, BENZ2616), one Husheep (17, sample accession SAMN13678651) and on Ovis ammon polii x Ovis aries cross (18, sample accession SAMN26012028)

All animals but animal 09 showed more than three class I alleles (Table 2). To minimize the chance that an allele had been missed in this animal, the RNA-Seq data from animal 09 were thoroughly reanalyzed with an extended reference database containing bovine and caprine class I alleles in addition and using two more rounds of BWA-MEM/cap3 analyses, but there was no evidence of an additional allele. To investigate the zygosity at the MHC region the *DRB1*-genotype was determined. Animal 09 was homozygous for the highly polymorphic *DRB1* gene. This strongly suggests a homozygous status for the MHC region, which in this animal contains one haplotype with 3 MHC class I alleles.

### Alleles identified

**Fig. 3:**
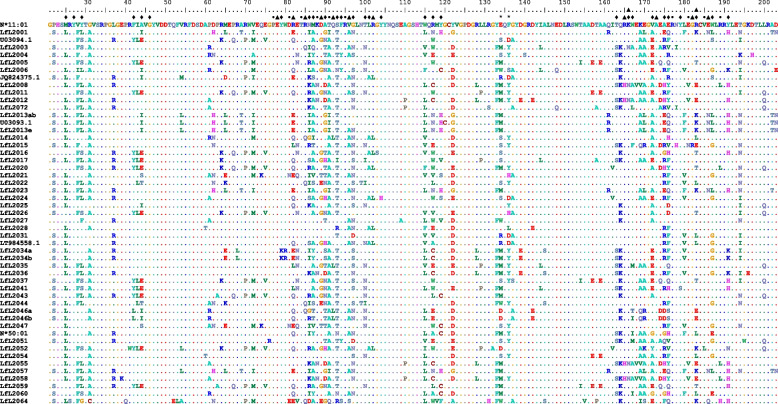
Amino acid sequences of MHC class I alleles derived from RNA-Seq data from 16 sheep. Alignment of ovine MHC class I amino acid sequences from 6 Texel x Scottish Blackface and 9 Merino sheep. Amino acids identical to allele N*11:01 are indicated by a dash. Positions are numbered according to IPD. Positions in contact to the antigen are indicated by diamonds. The 6 pockets are labeled by different colors

In the 15 animals, 51 MHC class I alleles (Tables 1 and 2, Supplementary file S1) were identified based on the sequence information of exons 2 and 3, which encode the α1 and α2 domains of the class I molecule spanning the antigen-binding groove (Fig. 3). From these 51 alleles, 45 were novel. The remaining six alleles were identical to NCBI database entries including two that were identical to IPD defined alleles (N*11:01 and N*50:01, Table 1). The nucleotide sequences of alleles LfL2031 and LT984558.1 were identical for over 500 bps (the 5-prime 247 bp and 3-prime 256 bp, compare Fig. 3) but were highly divergent (20% differences) at the region from nt position 248 to 353. This seems to be an obvious example for a gene conversion event (e.g. [32]). All derived amino acid sequences were different, i.e. no pair of translated mRNA sequences from this work was identical (Fig. 3). When including all IPD defined alleles in the comparison one allele, LfL2027 shows one nucleotide difference to N*02:01, but both alleles have identical derived amino acid sequences.

Fourteen alleles occurred in more than one of the 15 sheep, 4 of those are shared between the two different breeds. Since no relatives were included in the analysis haplotypes could not be derived with confidence. Assuming animal 09 as homozygous for MHC class I there are 3 alleles per haplotype in average.

Several MHC class I genes are transcribed per haplotype and virtually all individuals express more than two alleles. This complicates the estimation of the number of reads aligned to MHC class I for expression studies. To get a rough estimate of the relative transcription level for single alleles the number of aligned reads was determined at a region that differs between most alleles and allows allele-specific alignments. I used the number of reads aligned to the allele at nucleotide position 260 (according to IPD) to make sure that the great majority of aligned sequence reads is allele specific. Based on the number of reads at that position alleles were grouped in three categories (< 1000 ~ low, 1000–5000 ~ middle, > 5000 ~ high; Table 1).

The MHC class I molecule forms 6 pockets that have contact to the antigen (Fig. 3). When only the positions that have contact with the antigen were compared, two groups (group 1—LfL2001, N*50:03, LfL2013e, LfL2013a, U03093.1 and group 2—LfL2055, LfL2008, LfL2012, LfL2058, N*12:01, N*08:01, N*22:01) of alleles were found, that were identical, or close to identical at these positions with zero to 3 differences (from 31 amino acids) and some allele pairs exist with identical amino acids at the positions with contact to the antigen but with differences in the remaining protein (e.g. LfL2008 and LfL2055). This would be in concordance with low variable, non-classical MHC class I alleles. At some positions specific amino acids occur exclusively in putative non-classical alleles in this dataset (group 1: positions with contact of the antigen—p.D62H, p.M65L, p.A69T, p.T91I, p.Y122G, p.Q161R, p.E170L, p.L180F, p.E186N, p.AD222-223TN –, remaining sequence—p.R117N, p.A175E -; group 2: positions with contact of the antigen—p.K88N, p.E181V -, remaining sequence—p.A90D -). These positions correspond in part to positions where human non-classical class I genes show specific amino acids, e.g. 117 (human non-classical—W, G or V—*vs*. classical—I, R or M -).

### Matching of DinoMfRS derived MHC class I sequences to genomic sequence

To evaluate further applications and to confirm the reliability of DinoMfRS the method was extended to three sheep for which both RNA-Seq data and whole-genome shotgun sequencing (WGS) information were available from SRA database. The first was Benz2616, the Rambouillet sheep that was used for generating the ovine genome reference assembly ARS-UI_Ramb_v2.0 (NCBI annotation release 104, 2021/07/04). In a first step MHC class I alleles were identified from the RNA-Seq data. Nine class I alleles were derived (LfL2082-2090, Tables 1 and 2, Supplementary Fig. S1) and all of them matched to genomic sequences from Benz2616. Five alleles (LfL2082, LfL2083, LfL2087, LfL2088, LfL2089) matched 100% to the genome assembly (OAR20, Accession number JAEVFA010000127.1) (Table 3) and all but LfL2088 are annotated as MHC class I genes. The 4 remaining alleles (LfL2084, LfL2085, LfL2086, LfL2090) are not covered by the genome reference sequence but match 100% with sequences in two whole-genome contigs assembled from OAR_USU_Benz2616 (accession numbers PEKD01004038 and PEKD01004039) that were not assigned to a chromosome jet. According to the sequence-based criteria from the two other datasets allele LfL2085 would fit into the group 2 non-classical genes.

**Table 3:** Alignment of RNA-Seq derived MHC class I alleles with a genomic de novo assembly from the same individual for three animals

Rambouillet (BENZ2616) accession SAMN17575729; assembly GCF_016772045.1								
allel	exon 2			intron 2		exon 3		
	start	End	identity	length [bp]		start	end	identity
LfL2082	27177386	27177657	100%	196		27177853	27178129	100%
LfL2083	27748,145	27747874	100%	199		27747675	27747399	100%
*LfL2084*	*27664922*	*27664651*	*89%*	*197*		*27664454*	*27664178*	*91%*
*LfL2085*	*27707857*	*27707586*	*93%*	*200*		*27707386*	*27707110*	*93%*
*LfL2086*	*27707857*	*27707586*	*91%*	*200*		*27,707,386*	*27707110*	*92%*
LfL2087	26,983,972	26984243	100%	194		26984437	26984713	100%
LfL2088	27664922	27664651	100%	197		27664454	27664178	100%
LfL2089	27707857	27707586	100%	200		27707386	27707110	100%
*LfL2090*	*27707857*	*27707586*	*99%*	*200*		*27707386*	*27707110*	*98%*
HuSheep accession SAMN13678651; assembly ASM1117029v1								
allel	exon 2				intron 2	exon 3		
	start	End	identity		length [bp]	start	end	identity
LfL2091	30275282	30275011	100%		193	30274818	30274542	100%
LfL2092	30347015	30346744	100%		199	30346545	30346269	100%
LfL2093	30204669	30204397	100%		201	30204196	30203919	100%
*LfL2094*	*29123944*	*29124215*	*94%*		*271*	*29123944*	*29124215*	*95%*
LfL2015	29705916	29706187	100%		196	29706383	29706659	100%
*LfL2095*	*29421461*	*29421732*	*96%*		*195*	*29421927*	*29422203*	*89%*
LfL2096	20406404	20406675	100%		221	20406896	20407172	100%
Ovis ammon polii x Ovis ariesaccession SAMN26012028 assembly GCA_023701675.1								
allel	exon 2				intron 2	exon 3		
	start	End	identity		length [bp]	start	end	identity
LfL2097	34975557	34975828	100%		195	34976023	34976300	100%
*LfL2098*	*35749893*	*35749622*	*90%*		*197*	*35749425*	*35749149*	*90%*
*LfL2099*	*35749893*	*35749622*	*91%*		*197*	*35749425*	*35749149*	*91%*
LfL2100	35786189	35785918	100%		195	35785723	35785447	100%
LfL2101	35019521	35019791	100%		186	35019977	35020255	100%
*LfL2102*	*35786189*	*35785918*	*95%*		*195*	*35785723*	*35785555*	*92%*
LfL2103	35855847	35855576	100%		194	35855382	35855106	100%
*LfL2104*	*35855847*	*35855576*	*89%*		*194*	*35855382*	*35855106*	*95%*
*LfL2105*	*34975557*	*34975828*	*94%*		*271*	*34975557*	*34975828*	*94%*

Results from alignment of RNA-Seq derived MHC alleles against genomic assembly for animal BENZ2616, the Huheep and the Ovis ammon polii x Ovis aries cross. Exon 2 and 3 had been aligned separately, the start and end position according to the assembly numbering and the length of the intron are indicated

The second was a male Husheep [33]. Seven ovine MHC class I alleles were identified (Table 1 and Supplementary figure S2) using DinoMfRS. Six of these (LfL2091-LfL2096) were novel (Table 1 and 2). Alignment with the genomic assembly revealed a complete match for five alleles (Table 3). Alignment to the original SRA reads resulted in 100% identity for all 7 alleles (Supplementary table 1).

The third was from an Ovis ammon polii x Ovis aries cross and the DinoMfRS yielded 9 MHC class I alleles (Table 1 and Supplementary Fig. S3), all of which were novel. Four alleles had 100% identity to the corresponding genome assembly (Table 3) and all 9 alleles showed complete match to the a large number of original SRA reads (Supplementary table 1).

## Discussion

Information about MHC class I alleles is sparse in sheep, partly due to the lower research resources available compared e.g. to humans or cattle but also due to the complex haplotype and gene organization of the MHC. Less than 40 MHC class I alleles are officially assigned by the IPD database, compared to more than 20.000 in humans. This incomplete knowledge of allelic variation limits, among other issues, the development of typing systems and immunological studies. Therefore, new cost-efficient ways to expand the ovine class I allele panel are urgently sought.

MHC class I alleles are among the most polymorphic genes of all. For example, if we consider the region between nucleotide position 474 and 593 for the alleles found in this publication, the average number of nucleotide differences is 18.5 with a maximum number of 32/120 bp (15% and 27% differences, respectively) in a length range common for sequence reads from RNA-Seq experiments. This high degree of polymorphism combined with the unclear genomic organization of MHC class I genes makes the identification of new alleles in sheep very difficult.

DinoMfRS combines the alignment of RNA-Seq data to an incomplete MHC class I reference database with de novo reassembly of a subset of RNA-Seq reads (align—> extraction of RNA-Seq reads—> de novo reassemble) to get the information about new alleles. This strategy proved to be highly effective. From 18 animals 69 novel MHC class I alleles could be identified. This almost doubled the number of known ovine MHC class I alleles and will facilitate the detection of further alleles in future experiments. Only 4 alleles identified in this work are shared between breeds. This reflects the high genetic plasticity of MHC genes, which leads to population-specific allele sets.

A similar method, RAMHCIT, has been used to determine MHC alleles from RNA-Seq data in cattle [34]. A prerequisite for the use of RAMHCIT is the availability of a larger number of known alleles (as in the case of bovine MHC), since more divergent alleles cannot be identified, resulting in largely incomplete genotyping. RAMHCIT identifies novel alleles by using bowtie [35] for alignment with the -v3 option, which allows a maximum of 3 bp nucleotide differences from the reference sequence. This stringent value hinders the alignment of reads especially in the highly variable regions of MHC class I genes when the variability present in the population is not sufficiently covered by the reference library. The use of higher v-values in Bowtie results in a significant number of misaligned reads. A problem that was successfully overcome here by combining a less stringent alignment method with de novo assembly using stringent alignment conditions.

Due to the large number of alleles and polymorphic positions approaches for identifying MHC alleles carry the risk of artifacts, such as overlooking incorrectly recombined or assembled sequence regions. In the present work the risk for artifacts was minimized by: 1) Using the penalty option for unpaired reads in BWA-MEM. As a result, each 5' and 3' region was always covered by forward and reverse reads from one fragment (Supplementary Fig. 4). 2) Many alleles (including some very divergent alleles like LfL2011 or LfL2026 were independently present in several animals. 3) RNA and genomic sequence data were available for three sheep. In all three cases, the alleles obtained from the RNA data could be verified against the genomic data. Therefore, artefact variants can be virtually ruled out here. In particular, this also applies to the alleles LfL2031 and LT984558.1, which have long identical 5' and 3' sequence stretches. An artificial recombination or misalignment can almost be excluded, especially since they do not occur together in one animal but were found independently in two animals of different breed.

DinoMfRS enables resource-efficient expansion of the MHC class I allele database by using existing data from NGS experiments. RNA-Seq data are used for a variety of different applications, from quantification of expression to analysis of splice variants. As the cost of NGS technology continues to decrease, the number of available RNA-Seq datasets in farm animals is rapidly increasing. However, inference of class I alleles from these datasets in sheep has been largely unsuccessful because the methods used rely on strict alignment algorithms, which by their nature do not allow alignment of highly divergent sequence reads to genomic or transcriptomic reference sequences. The approach developed here overcomes these obstacles by combining the alignment to a reference sequence with a de novo alignment approach and enables the identification of MHC class I alleles from ovine RNA-Seq data despite the high degree of sequence differences.

Using DinoMfRS, all expressed alleles can be identified. While methods using specific PCR primers run the risk of missing alleles that show variation in the primer sequence region, the use of RNA-Seq data enables unbiased identification of all alleles. This is a major advantage, especially for highly polymorphic gene regions such as the MHC. In addition, MHC class I molecules are basically expressed on the surface of almost all cells that contain a nucleus and this, in combination with the high expression level greatly facilitates their identification even in RNA-Seq datasets with a limited number of reads. In addition, DinoMfRS can be easily extended to other genes such as MHC class II and allows the study of expression levels.

The efficiency of DinoMfRS increases with the number of known alleles. When little information is available about the allelic spectrum the identification of the first novel alleles using DinoMfRS is time consuming since several steps are necessary. With increasing completeness of the reference database the method speeds up, since more and more alleles can be determined by one BWA-MEM run. When the allelic spectrum is sufficiently covered by the reference database the method can be used for low or medium throughput high resolution genotyping of MHC e.g., using RAMHCIT. Several techniques have been developed for high-resolution genotyping of MHC alleles from short sequence reads, most of them for the HLA with its high information density available [36]. The approaches can be divided into two groups: the de novo assembly and the alignment approach, in which short sequence sequences are aligned to known reference allele sequences. Both have their disadvantages [36]. DinoMfRS combines the advantages of both approaches, and generating RNA-Seq data from individuals combined with DinoMfRS may be an alternative to current genotyping methods for MHC genes, which are complex and expensive.

When RNA-Seq data are available for the animal whose DNA was sequenced to build a genomic sequence, DinoMfRS can greatly improve annotation of the MHC region, as demonstrated here for the current reference sheep genome. Although a high-quality reference sheep genome is available, annotation of the MHC region is incomplete. Using DinoMfRS derived alleles from Benz2616 RNA-Seq data, the annotation of MHC class I alleles in the genome sequence was reviewed. At least one MHC class I allele was present in the reference sequence but not annotated, some MHC class I gen-containing contigs have not yet been mapped to the ovine chromosome sequence, and the current sheep genome release contains sequences from two different haplotypes. Based on whole-genome contig data in combination with DinoMfRS it could be shown that alleles LfL2082, LfL2087, and LfL2088 likely comprise one haplotype and alleles LfL2083, LfL2084, LfL2085, LfL2086, and LfL2090 comprise the second. With this approach DinoMfRS can support the accurate diploid genome assembly for the MHC region.

DinoMfRS can contribute to a number of further research fields. One is the clarification of the evolutionary mechanisms driving the high variability of MHC genes. The evolution of MHC class I genes of the family Bovidae remains largely obscure up to now because neither the assignment of alleles to individual gene loci nor the allelic spectrum is sufficiently known. Completion of the allelic spectrum also facilitates the annotation of genes in genomic sequences and can thus contribute in two ways to elucidating the evolutionary mechanisms of the MHC and related aspects such as mate choice influenced by the MHC. DinoMfRS can help to overcome the problems in gene expression studies that include MHC class I genes. It enables the analysis of all expressed MHC class I alleles individually, overcoming methodological problems in identifying differentially expressed MHC genes in gene expression profiling. Further, it will be useful for identifying expressed alleles in other gene-families that show different copy numbers per chromosome or are highly polymorphic (e.g. odorant receptors [37]).

Finally, as is already the case in humans and experimental animals, the identification of pathogen antigens bound to MHC and their recognition will become important in the future for understanding disease resistance in livestock. The process of assigning each ligand to its presenting MHC molecule is a critical step in the analysis of MHC ligand data [38]. The completion of the MHC class I allelic spectrum will be a prerequisite for wet-lab and bioinformatics analyses of the immunopeptidome in sheep.

## Conclusion

In sheep, only a few MHC class I alleles have been described so far. With the method developed here, divergent ovine class I alleles can be extracted from existing RNA-Seq data for the first time, allowing the class I allele spectrum to be rapidly extended. Other applications for DinoMfRS include genotyping, annotation of MHC genes and expression studies for highly polymorphic genes.

## Materials and methods

The strategy developed here is based on alignment of NGS reads against a custom-built reference sequence database followed by a de novo assembly approach. DinoMfRS combines alignment of RNA-Seq data to an MHC class I reference database with de novo reassembly of a subset of RNA-Seq reads.

### Next generation sequencing data sets

I used two different ovine RNA-Seq data sets obtained from the FAANG database as hosted by the European Nucleotide Archive [ENA, 31]. Both sets provide cleaned FASTQ reads from paired-end sequencing. The first one was from study PRJEB19199. These data were generated from Texel x Scottish Blackface (TExSC) crossbred individuals using 125 bp long, paired-end reads from polyA captured cDNA libraries [39]. The second set was generated within an Irish *Fasciola hepatica* project (PRJNA291172) that used Merino (ME) sheep [40]. The RNA-Seq libraries from this project were prepared in the 2 × 100 bp format using polyA captured DNA. Clean fastq files were downloaded from the Sequence Read Archive (SRA) at the ENA server to the local harddisk. In total, data from 15 sheep, 6 Texel x Scottish Blackface (animal 01–06, Accession Numbers SAMEA6256918, SAMEA6273418, SAMEA6265168, SAMEA5181418, SAMEA5208418, SAMEA5219668) and 9 Merino (animal 07–15, Accession Numbers SAMN03940431, SAMN03940432, SAMN03940433, SAMN03940440, SAMN03940441, SAMN03940449, SAMN03940450, SAMN03940451, SAMN03940453) from these projects were included in the analysis.

Additional datasets from animals, for each of which RNA-Seq and genome sequence data were available, were used to confirm the method. One RNA-Seq dataset (2 × 101 bp format) from a female Rambouillet sheep, Benz2616 was extracted from the FAANG Data Coordination Centre (run SRR6651987). The same animal, Benz2616 was used to generate the whole-genome shotgun sequences for the current annotation of the ovine genome ARS-UI_Ramb_v2.0 (NCBI annotation release 104, 2021/07/04). The ovine whole-genome contig database was accessed at NCBI (by 2021/08/01). A second one was from a male Husheep [33] (biosample accession SAMN13678651; RNA-Seq data accession SRR10821773, 150 bp read length, paired, 11.5 G; genomic de novo assembly accession ASM1117029v1). A third one was from a cross of Ovis ammon polii x Ovis aries (biosample accession SAMN26012028; RNA-Seq data accession SRR19412708, 150 bp read length, paired-end, 9.4 G; genomic de novo assembly accession GCA_023701675.1).

### Initial MHC class I reference database

To obtain the MHC class I alleles from RNA-Seq data as complete as possible the reference database is of crucial importance. Ovine Class I alleles were extracted from the ImmunoPolymorphism Database [16]. Further sequences were identified by BLAST searches of the NCBI nucleotide collection (consisting of GenBank, EMBL, DDBJ, PDB and RefSeq sequences, [41]) and added to the collection.

All sequences were aligned using clustal w and trimmed to a region spanning exon 2 and 3. This restriction was chosen since all polymorphism that determine the binding affinity to the antigenic peptides are encoded by these two exons and most published sequences cover this region. From pairs of alleles with < 5 bp differences one was deleted to reduce redundancy. The remaining sequences formed the initial reference library. Editing of sequence alignments, translation into amino acid sequences and preparing of sequence-graphs was done with BIOEDIT (v. 7.1).

### Mapping of RNA-Seq reads to the initial reference database

The RNA-Seq reads from the 15 sheep were aligned to the MHC class I reference database using the BWA-MEM (bwa-0.7.12) algorithm [42] using the following parameters (-A 1 –B 4 -w 100 -L 5). For the final round of alignments against the updated database the parameters were adopted to higher stringency condition (-A 2 -B 5 -w 30 -L 2) to avoid mapping of dissimilar sequence fragments.

### Definition and refinement of MHC class I alleles

BWA-MEM alignments were visually inspected and evaluated using UGENE Assembly Browser [43] (ver. 33) and/or IGV [44] (ver. 2.2.10). Reading and editing of the resulting files in BAM format was done using samtools (ver. 0.1.19). When a consensus sequence of an alignment was identical to a known allele, it was kept as an allele for the individual. Alignments that resulted in hereto unknown alleles and ambiguous consensus sequences were further analyzed if they consists of more than 500 aligned reads over a length of > 400 bp. Reads from these alignments were extracted to a single fasta-file (including only paired reads) reanalyzed by *de-novo* assembly using cap3 [45] applying stringent conditions (-p 99 -i 30 -j 60 -o 40 -s 300). Resulting unambiguous consensus sequences were added to the individual reference library. Finally, all alleles were confirmed by a BWA-MEM run using the final library containing the complete set of alleles found in the individual.

### Combined analysis of RNA-Seq and WGS data

Both, RNA-Seq and WGS data from OAR_USU_Benz2616 were used to explore the possible combination of both data types. MHC class I alleles derived by DinoMfRS (based on Run SRR6651987) were used as a query to blast the whole-genome contigs from the *Ovis aries* Rambouillet genome sequencing and assembly project to proof the identity of the RNA derived alleles with genome-sequences. The same approach was used for the Husheep and the Ovis ammon polii x Ovis aries cross. To ensure that alleles not captured by the de novo assemblies were real, they were aligned against the original genomic reads (sequel II, pacbio, accession SRR19412709 and SRX15467208 for the Husheep and the Ovis ammon polii x Ovis aries cross, respectiv).

## Supplementary Information

**Additional file 1: Supplementary figure S1.** Amino acid sequences of MHC class I alleles derived from RNA-Seq data from Benz2616.

**Additional file 2: Supplementary figure S2.** Amino acid sequences of MHC class I alleles derived from RNA-Seq data from Hu Sheep.

**Additional file 3: Supplementary figure S3.** Amino acid sequences of MHC class I alleles derived from RNA-Seq data from Ovis ammon x and Ovis aries cross.

**Additional file 4: Supplementary table S1.** Read accessions from WGS reads containing the alleles identified by DinoMfRS from Husheep and the ammon x aries cross. The alleles are given and the accession of one of the reads that contain the complete allele with 100% identity.

**Additional file 5: Supplementary figure S4.** View of the final alignment for allele LfL2091 using the Integrative Genomics Viewer version 2.2.11 with the „view as pairs” option. Forward (red) and reverse (blue) sequence from a fragment are connected with a line indicating the intervening sequence.

**Additional file 6: Supplementary file S1.** Sequence file containing all new ovine MHC class I sequences in fasta format.

## Data Availability

The novel ovine MHC class I sequences are available in GenBank/EMBL-EBI DNA databases under the Accession Numbers OL628766, OL628767, OL628768, OL628769, OL628770, OL628771, OL628772, OL628773, OL628774, OL628775, OL628776, OL628777, OL628778, OL628779, OL628780, OL628781, OL628782, OL628783, OL628784, OL628785, OL628786, OL628787, OL628788, OL628789, OL628790, OL628791, OL628792, OL628793, OL628794, OL628795, OL628796, OL628797, OL628798, OL628799, OL628800, OL628801, OL628802, OL628803, OL628804, OL628805, OL628806, OL628807, OL628808, OL628809, OL628810, OL628811, OL628812, OL628813, OL628814, OL628815, OL628816, OL628817, OL628818, OL628819.

## Abbreviations

CDS: Coding DNA sequence
DinoMfRS: Discovery of novel MHC class I alleles from RNA-Seq data
ENA: European Nucleotide Archive
HLA: Human Leukocyte Antigen
MHC: Major Histocompatibility Complex
NGS: Next-Generation Sequencing
PCR: Polymerase Chain Reaction
RNA-Seq: NGS RNA sequencing
Tcr: T cell receptor
WGS: Whole-genome shotgun sequence data

## References

[1] Rötzschke O, Falk K, Deres K, Schild H, Norda M, Metzger J, Jung G, Rammensee H-G (1990). Isolation and analysis of naturally processed viral peptides as recognized by cytotoxic T cells. Nature.

[2] Madden DR (1995). The Three-Dimensional Structure of Peptide-MHC Complexes. Annu Rev Immunol.

[3] Mariuzza R, Li Y (2014). Structural Basis for Recognition of Cellular and Viral Ligands by NK Cell Receptors. Front Immunol.

[4] Istrail S, Florea L, Halldórsson BV, Kohlbacher O, Schwartz RS, Yap VB, Yewdell JW, Hoffman SL (2004). Comparative immunopeptidomics of humans and their pathogens. Proc Natl Acad Sci USA.

[5] Matzaraki V, Kumar V, Wijmenga C, Zhernakova A (2017). The MHC locus and genetic susceptibility to autoimmune and infectious diseases. Genome Biol.

[6] Dallmann-Sauer M, Fava VM, Gzara C, Orlova M, Van Thuc N, Thai VH, Alcaïs A, Abel L, Cobat A, Schurr E (2020). The complex pattern of genetic associations of leprosy with HLA class I and class II alleles can be reduced to four amino acid positions. PLoS Pathog.

[7] Lohr CE, Sporer KRB, Brigham KA, Pavliscak LA, Mason MM, Borgman A, Ruggiero VJ, Taxis TM, Bartlett PC, Droscha CJ (2022). Phenotypic Selection of Dairy Cattle Infected with Bovine Leukemia Virus Demonstrates Immunogenetic Resilience through NGS-Based Genotyping of BoLA MHC Class II Genes. Pathogens.

[8] Larruskain A, Minguijón E, García-Etxebarria K, Moreno B, Arostegui I, Juste RA, Jugo BM (2010). MHC class II DRB1 gene polymorphism in the pathogenesis of Maedi-Visna and pulmonary adenocarcinoma viral diseases in sheep. Immunogenet.

[9] Wallny H-J, Avila D, Hunt LG, Powell TJ, Riegert P, Salomonsen J, Skjødt K, Vainio O, Vilbois F, Wiles MV (2006). Peptide motifs of the single dominantly expressed class I molecule explain the striking MHC-determined response to Rous sarcoma virus in chickens. Proc Natl Acad Sci.

[10] Buitkamp J, Filmether P, Stear MJ, Epplen JT (1996). Class I and class II *major histocompatibility complex* alleles are associated with faecal egg counts following natural, predominantly *Ostertagia circumcincta* infection. Parasitol Res.

[11] Schlosstein L, Terasaki PI, Bluestone R, Pearson CM (1973). High association of an HL-A antigen, W27, with ankylosing spondylitis. N Engl J Med.

[12] Eyre S, Bowes J, Diogo D, Lee A, Barton A, Martin P, Zhernakova A, Stahl E, Viatte S, McAllister K (2012). High-density genetic mapping identifies new susceptibility loci for rheumatoid arthritis. Nature Genet.

[13] Nejentsev S, Howson JM, Walker NM, Szeszko J, Field SF, Stevens HE, Reynolds P, Hardy M, King E, Masters J (2007). Localization of type 1 diabetes susceptibility to the MHC class I genes HLA-B and HLA-A. Nature.

[14] IPD-IMGT/HLA Release 3.50: https://www.ebi.ac.uk/ipd/imgt/hla/stats.html.

[15] Parham P, Adams EJ, Arnett KL (1995). The origins of HLA-A, B, C polymorphism. Immunol Rev.

[16] Maccari G, Robinson J, Ballingall K, Guethlein LA, Grimholt U, Kaufman J, Ho C-S, de Groot NG, Flicek P, Bontrop RE (2017). IPD-MHC 2.0: an improved inter-species database for the study of the major histocompatibility complex. Nucleic Acids Res.

[17] IPD-MHC Release 3.9.0.1 https://www.ebi.ac.uk/ipd/mhc/group/OLA/species/

[18] Ballingall KT, Miltiadou D, Chai ZW, McLean K, Rocchi M, Yaga R, McKeever DJ (2008). Genetic and proteomic analysis of the MHC class I repertoire from four ovine haplotypes. Immunogenet.

[19] Miltiadou D, Ballingall KT, Ellis SA, Russell GC, McKeever DJ (2005). Haplotype characterization of transcribed ovine major histocompatibility complex (MHC) class I genes. Immunogenet.

[20] Subramaniam SN, Morgan EF, Wetherall JD, Stear MJ, Groth DM (2015). A comprehensive mapping of the structure and gene organisation in the sheep MHC class I region. BMC Genomics.

[21] Ellis SA, Holmes EC, Staines KA, Smith KB, Stear MJ, McKeever DJ, MacHugh ND, Morrison WI (1999). Variation in the number of expressed MHC genes in different cattle class I haplotypes. Immunogenet.

[22] Schwartz JC, Hammond JA (2015). The assembly and characterisation of two structurally distinct cattle MHC class I haplotypes point to the mechanisms driving diversity. Immunogenet.

[23] Schwartz JC, Maccari G, Heimeier D, Hammond JA (2022). Highly-contiguous bovine genomes underpin accurate functional analyses and updated nomenclature of MHC class I. HLA.

[24] Wiseman RW, Karl JA, Bimber BN, O'Leary CE, Lank SM, Tuscher JJ, Detmer AM, Bouffard P, Levenkova N, Turcotte CL (2009). Major histocompatibility complex genotyping with massively parallel pyrosequencing. Nat Med.

[25] Babik W (2010). Methods for MHC genotyping in non-model vertebrates. Mol Ecol Resour.

[26] Promerova M, Babik W, Bryja J, Albrecht T, Stuglik M, Radwan J (2012). Evaluation of two approaches to genotyping major histocompatibility complex class I in a passerine-CE-SSCP and 454 pyrosequencing. Mol Ecol Resour.

[27] Schwaiger FW, Buitkamp J, Weyers E, Epplen JT (1993). Typing of artiodactyl *MHC-DRB* genes with the help of intronic simple repeated DNA sequences. Mol Ecol.

[28] Ammer H, Schwaiger F-W, Kammerbauer C, Gomolka M, Arriens A, Lazary S, Epplen JT (1992). Exonic polymorphism versus intronic simple repeat hypervariability in *MHC-DRB*-genes. Immunogenet.

[29] Sigurdardottir S, Borsch C, Gustafsson K, Andersson L (1991). Cloning and sequence analysis of 14 DRB alleles of the bovine major histocompatibility complex by using the polymerase chain reaction. Anim Genet.

[30] Vasoya D, Law A, Motta P, Yu M, Muwonge A, Cook E, Li X, Bryson K, MacCallam A, Sitt T (2016). Rapid identification of bovine MHC I haplotypes in genetically divergent cattle populations using next-generation sequencing. Immunogenet.

[31] ENA, European Nucleotide Archive: https://www.ebi.ac.uk/ena

[32] Zangenberg G, Huang M-M, Arnheim N, Erlich H (1995). New HLA–DPB1 alleles generated by interallelic gene conversion detected by analysis of sperm. Nature Genet.

[33] Li R, Yang P, Li M, Fang W, Yue X, Nanaei HA, Gan S, Du D, Cai Y, Dai X (2020). A Hu sheep genome with the first ovine Y chromosome reveal introgression history after sheep domestication. Sci China Life Sci.

[34] Demasius W, Weikard R, Hadlich F, Buitkamp J, Kuhn C (2016). A novel RNAseq-assisted method for MHC class I genotyping in a non-model species applied to a lethal vaccination-induced alloimmune disease. BMC Genomics.

[35] Langmead B, Trapnell C, Pop M, Salzberg SL (2009). Ultrafast and memory-efficient alignment of short DNA sequences to the human genome. Genome Biol.

[36] Ka S, Lee S, Hong J, Cho Y, Sung J, Kim H-N, Kim H-L, Jung J (2017). HLAscan: genotyping of the HLA region using next-generation sequencing data. BMC Bioinformatics.

[37] Buck L, Axel R (1991). A novel multigene family may encode odorant receptors: a molecular basis for odor recognition. Cell.

[38] Alvarez B, Reynisson B, Barra C, Buus S, Ternette N, Connelley T, Andreatta M, Nielsen M (2019). NNAlign_MA; MHC Peptidome Deconvolution for Accurate MHC Binding Motif Characterization and Improved T-cell Epitope Predictions. Mol Cell Proteomics.

[39] Clark EL, Bush SJ, McCulloch MEB, Farquhar IL, Young R, Lefevre L, Pridans C, Tsang HG, Wu C, Afrasiabi C (2017). A high resolution atlas of gene expression in the domestic sheep (Ovis aries). PLoS Genet.

[40] Fu Y, Chryssafidis AL, Browne JA, O'Sullivan J, McGettigan PA, Mulcahy G (2016). Transcriptomic Study on Ovine Immune Responses to Fasciola hepatica Infection. PLoS Negl Trop Dis.

[41] BLAST W: Web BLAST: https://blast.ncbi.nlm.nih.gov/Blast.cgi.

[42] Li H. Aligning sequence reads, clone sequences and assembly contigs with BWA-MEM. arXiv 2013:1303.3997.

[43] Okonechnikov K, Golosova O, Fursov M, team U (2012). Unipro UGENE: a unified bioinformatics toolkit. Bioinformatics.

[44] Thorvaldsdottir H, Robinson JT, Mesirov JP (2013). Integrative Genomics Viewer (IGV): high-performance genomics data visualization and exploration. Brief Bioinform.

[45] Huang X, Madan A (1999). CAP3: A DNA sequence assembly program. Genome Res.

