# Supplementary material for: Uncovering novel MHC alleles from RNA-Seq data: expanding the spectrum of MHC class I alleles in sheep
Source: BMC Genom Data. 2023 Jan 3;24:1. doi: 10.1186/s12863-022-01102-5 (PMC9809118; doi:10.1186/s12863-022-01102-5)
Supplement: Supplementary file 4 — Additional file 4: Supplementary table S1. Read accessions from WGS reads containing the alleles identified by DinoMfRS from Husheep and the ammon x aries cross. The alleles are given and the accession of one of the reads that contain the complete allele with 100% identity. [file 12863_2022_1102_MOESM4_ESM.docx]

**Supplementary Table 1:** **Read accessions from wgs reads containing the alleles identified by DinoMfRS**

Husheep

| Allel | Read accession  SRA:SRR10821772, | Ident |
| --- | --- | --- |
| LfL2091 | 546842966,2 | 100% |
| LfL2092 | 538848132,2 | 100% |
| LfL2093 | 533333596,1 | 100% |
| LfL2094 | 552171508,1 | 100% |
| LfL2015 | 552577798,1 | 100% |
| LfL2095 | 552171508,1 | 100% |
| LfL2096 | 543342521,1 | 100% |

Ovis ammon polii x Ovis aries cross

| Allel | Read accession  SRA:SRR19412709, | Ident |
| --- | --- | --- |
| LfL2097 | 5538955,1 | 100% |
| LfL2098 | 6466210,1 | 100% |
| LfL2099 | 6114709,1 | 100% |
| LfL2100 | 6435663,1 | 100% |
| LfL2101 | [3883351.1](https://www.ncbi.nlm.nih.gov/Traces/sra/sra.cgi?run=SRR19412709.3883351.1&RID=HK86BRAS016&display=reads) | 100% |
| LfL2102 | 5130770,1 | 100% |
| LfL2103 | 5478450,1 | 100% |
| LfL2104 | 6018547,1 | 100% |
| LfL2105 | 5553871,1 | 100% |
