# Supplementary figures and images for: Uncovering novel MHC alleles from RNA-Seq data: expanding the spectrum of MHC class I alleles in sheep
Source: BMC Genom Data. 2023 Jan 3;24:1. doi: 10.1186/s12863-022-01102-5 (PMC9809118; doi:10.1186/s12863-022-01102-5)

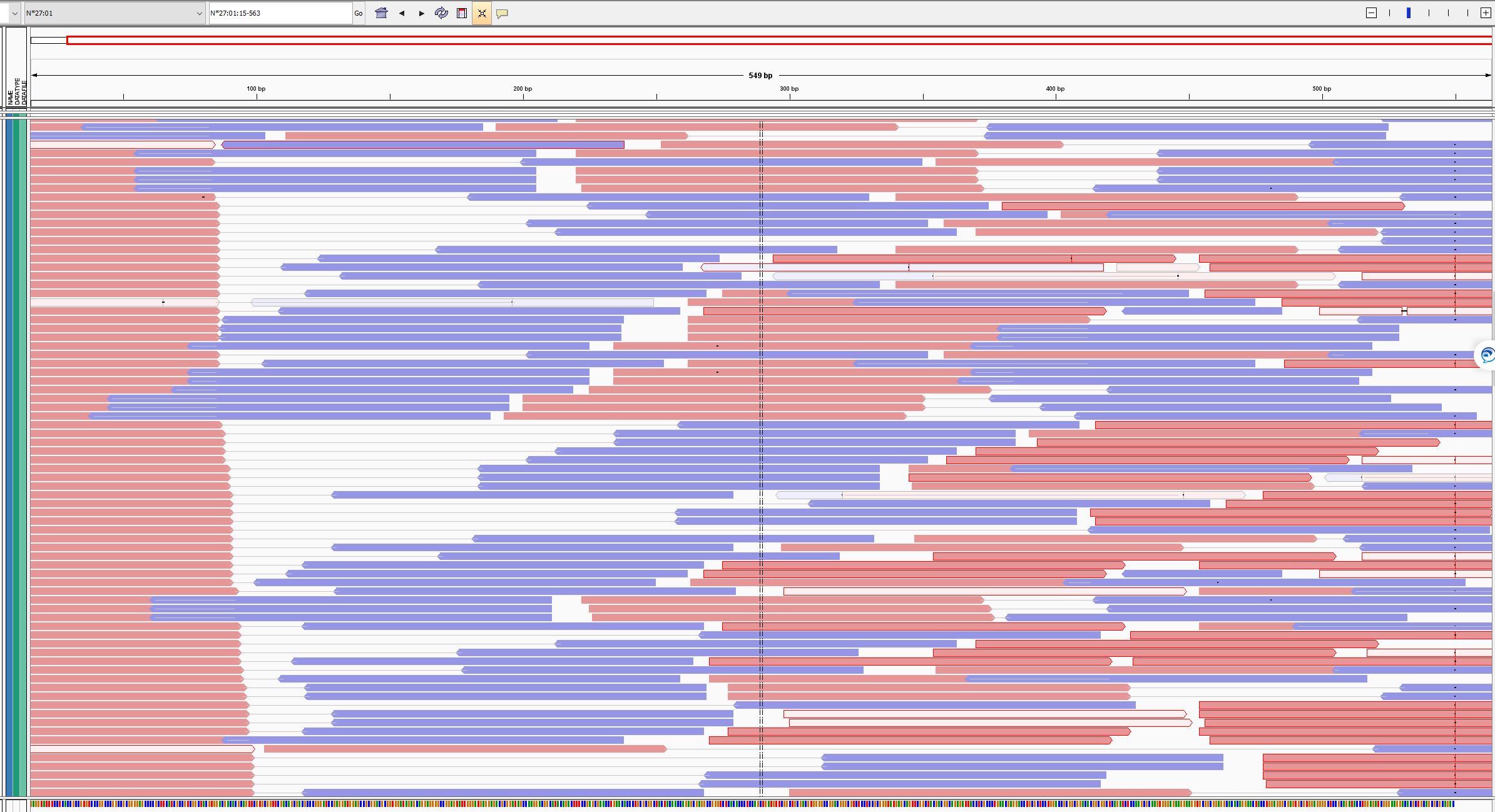

Supplement: Supplementary file 5 — Additional file 5: Supplementary figure S4. View of the final alignment for allele LfL2091 using the Integrative Genomics Viewer version 2.2.11 with the „view as pairs” option. Forward (red) and reverse (blue) sequence from a fragment are connected with a line indicating the intervening sequence. [file 12863_2022_1102_MOESM5_ESM.jpg]
