# Supplementary material for: Uncovering novel MHC alleles from RNA-Seq data: expanding the spectrum of MHC class I alleles in sheep
Source: BMC Genom Data. 2023 Jan 3;24:1. doi: 10.1186/s12863-022-01102-5 (PMC9809118; doi:10.1186/s12863-022-01102-5)
Supplement: Supplementary file 1 — Additional file 1: Supplementary figure S1. Amino acid sequences of MHC class I alleles derived from RNA-Seq data from Benz2616. [file 12863_2022_1102_MOESM1_ESM.pdf]

```
                  30        40        50        60        70        80        90       100       110       120
          ....|....|....|....|....|....|....|....|....|....|....|....|....|....|....|....|....|....|....|....|
              ♦ ♦ ♦              ♦        ♦♦                        ♦♦ ♦♦  ♦♦  ♦  ♦♦  ♦  ♦   ♦          ♦ ♦ ♦
N*11:01   GPHSMRYVYTGVSRPGLGEPRFIAVGYVDDTQFVRFDSDAPDPRMEPRARWVEQEGPEYWDRETRNMKDATQSFRVGLNTLRGYYNQSEAGSHTWQRMYG
LfL2082   .S..L..F..A..............................N...................LN..RT...A.T..AN..N..............V.E...
LfL2083   .S..............R.......................A...........................T.........N........A.......L...
LfL2084   .S..L...........R......S....................E..L..........KR.EN..IY..SA.TLG....................V.V...
LfL2085   .S..L..FL.A.....R...............................................KAN.DA.T...N.................L.C...
LfL2086   .S.....FS.A..........YLE.....................................Q...RT..TA.A......................L.C...
LfL2087   .S..L..FL.A.....R............................................Q..QGT..SALT..AN.................L.E...
LfL2088   .S..L..FG.A............S................L.....................N..RV...A.T..AN.................V.V...
LfL2089   ....L.....A..................................................Q...IY...A.T...N.................L.C.C.
LfL2090   ....L.....A.....R............................................Q...IY...A.T...N.................L.A.C.

                 130       140       150       160       170       180       190       200
          ....|....|....|....|....|....|....|....|....|....|....|....|....|....|....|....|...
                       ♦ ♦      ♦♦                   ♦  ♦♦    ♦  ♦♦  ♦   ♦   ♦
N*11:01   CYVGPDGRLLRGYEQFGYDGRDYIALNEDLRSWTAADTAAQITQRKWEKEGVAEAERNYLEGRCVEWLRRYLETGKDTLLRAD
LfL2082   .D.............S.S........................SK..F.QR.A.DRV.H..NRE...G......N.........
LfL2083   .D..............Y..........................K.......A...D.......................Q...
LfL2084   .D..........FW.SA.........................SK.......E..RF...V......G...K..I.........
LfL2085   .D.....L....FM............................SK.NAVVA.A..GH....V.E...S...H............
LfL2086   .D......P...FM.........L..................SK..M.AA.E..RF......T................Q...
LfL2087   .E........S..............................V...NA.AA.E.ARV.I....K....................
LfL2088   .D................A....E..................SK..L.......ARV.I.......G................
LfL2089   .D..........FM............................SK..I.AA.G..GH...F..L...E................
LfL2090   .D..........FM............................SK..I.AA.G..GH...F..L...E................
```
