# Supplementary material for: Uncovering novel MHC alleles from RNA-Seq data: expanding the spectrum of MHC class I alleles in sheep
Source: BMC Genom Data. 2023 Jan 3;24:1. doi: 10.1186/s12863-022-01102-5 (PMC9809118; doi:10.1186/s12863-022-01102-5)
Supplement: Supplementary file 2 — Additional file 2: Supplementary figure S2. Amino acid sequences of MHC class I alleles derived from RNA-Seq data from Hu Sheep. [file 12863_2022_1102_MOESM2_ESM.pdf]

```
                   30        40        50        60        70        80        90       100       110       120
           ....|....|....|....|....|....|....|....|....|....|....|....|....|....|....|....|....|....|....|....|
               ♦ ♦ ♦              ♦         ♦♦                        ♦♦ ♦♦  ♦♦  ♦  ♦♦  ♦  ♦   ♦              ♦ ♦ ♦
N*11:01    GPHSMRYVYTGVSRPGLGEPRFIAVGYVDDTQFVRFDSDAPDPRMEPRARWVEQEGPEYWDRETRNMKDATQSFRVGLNTLRGYYNQSEAGSHTWQRMYG
LfL2091    .S.....FS.A..........YLE...................K..Q..P.M.........Q...RA.GNGG.T...N...AL............I.W...
LfL2092    .S..L..FL.A.....R.......................L.....................N..RV..T..T.......................L.W...
LfL2093    .S..L..FL.A............I.........L.......H..L...T..I.........E...IA..GI.T..AN...................L.N.H.
LfL2094    ....L.....A............................T.....................Q...KA.GHA.I..AN...AL............V.E...
LfL2015    .S..L..F..A..............................N...................LN..RT...A.T..AN..N.................V.E...
LfL2095    ....L.....A.............................RN.........A.........Q..QGT...A.T..AN...AL............V.E...
LfL2096    .S..LS.FG.C........Q...........ELA.......N.......P.M........EEV.QDA.EGQ.RS.S.....................L.WVF.

                  130       140       150       160       170       180       190       200
           ....|....|....|....|....|....|....|....|....|....|....|....|....|....|....|....|...
                        ♦ ♦      ♦♦                  ♦  ♦♦    ♦  ♦♦  ♦   ♦   ♦   ♦
N*11:01    CYVGPDGRLLRGYEQFGYDGRDYIALNEDLRSWTAADTAAQITQRKWEKEGVAEAERNYLEGRCVEWLRRYLETGKDTLLRAD
LfL2091    ............FM......................E.E....K....AA.E.Q.Q......T................Q...
LfL2092    .D..........FM.........L..............A.....K.......A...GW.....E....................
LfL2093    .G..........FM..................................AL.A..EQ...F..K..NL...H..N.......SN
LfL2094    .D..........FM.Y.........................V.........A..........E...S.......N.........
LfL2015    .D...........S.Y...........................SK..F.QR.A.DRV.H..NRE...G.......N........E
LfL2095    .D..........FM.Y.........................V.........A..........E...S..................
LfL2096    .A........H.FW..A.......S.........V..P.....K....IS.E..FQ.....VK..Q..H.H............
```
