# Supplementary material for: Uncovering novel MHC alleles from RNA-Seq data: expanding the spectrum of MHC class I alleles in sheep
Source: BMC Genom Data. 2023 Jan 3;24:1. doi: 10.1186/s12863-022-01102-5 (PMC9809118; doi:10.1186/s12863-022-01102-5)
Supplement: Supplementary file 3 — Additional file 3: Supplementary figure S3. Amino acid sequences of MHC class I alleles derived from RNA-Seq data from Ovis ammon x and Ovis aries cross. [file 12863_2022_1102_MOESM3_ESM.pdf]

```
                  30        40        50        60        70        80        90       100       110       120
          ....|....|....|....|....|....|....|....|....|....|....|....|....|....|....|....|....|....|....|....|
N*11:01   GPHSMRYVYTGVSRPGLGEPRFIAVGYVDDTQFVRFDSDAPDPRMEPRARWVEQEGPEYWDRETRNMKDATQSFRVGLNTLRGYYNQSEAGSHTWQRMYG
LfL2097   .S..L..FL.A.............................RN.........A.........Q...SA.GHA....AN.................L.E...
LfL2098   .S..L.....A............S................AN..E........K......NEQ..IV.TTA.T...D.................L.C...
LfL2099   .S..L..FS.A...........LT.................N..K...................QIS.ENA.T...N.................L.C...
LfL2100   .S..L.....A.....R.....................E.RN.........A.........Q...MA...A.N...N.................V.A.C.
LfL2101   .S..LS.FG.C......................A.......N.......P.M........EEM..DA.K.Q.RL.S....M..F..E...V...S.WVFA
LfL2102   .S..L..FF.A.....R................................................RA..TA.I...N.................L.L.S.
LfL2103   .S..L..F..A.....R...........................E.................N..IY..TA.I...N...AL............V.E...
LfL2104   .S..L..FL.A.....R.......................R.......T..I.........E...IA..GI.T..AN.................L.L.F.
LfL2105   .S..L..FL.A.....R................L.......H..L................E...IA..GI.T..AN.................L.N.H.

                 130       140       150       160       170       180       190       200
          ....|....|....|....|....|....|....|....|....|....|....|....|....|....|....|....|...
N*11:01   CYVGPDGRLLRGYEQFGYDGRDYIALNEDLRSWTAADTAAQITQRKWEKEGVAEAERNYLEGRCVEWLRRYLETGKDTLLRAD
LfL2097   .E.......R......A......L.................V...NA.AA.D.ARV.I....K...................G
LfL2098   .D.....................L..................SK..F.AA.E...Q.................I.....Q...
LfL2099   .D.....................L..................SK.......A............L..................
LfL2100   .D....W.....FM.Y.......L.............A....SK..I.AA.G..GH...F..L...M......K.....Q...
LfL2101   .V..........IW.TA...A...S..................K....IS.E..FQ......K..Q..H.H............
LfL2102   .............S.........L...D..............SK..T.AA.G.DG.....V.E..G.......K.........
LfL2103   .D...........D.Y...........Q.......................A...H.........G.......I.........
LfL2104   .D.....................L..................SK.......A...H......E..G.......I.....Q...
LfL2105   .G..........FM..........................R.......AL.A..EQ...F..K..NL...H..N.......TN
```
